# Modular, Antibody-free Time-Resolved LRET Kinase Assay Enabled by Quantum Dots and Tb^3+^-sensitizing Peptides

**DOI:** 10.1038/srep28971

**Published:** 2016-07-18

**Authors:** Wei Cui, Laurie L. Parker

**Affiliations:** 1 Department of Biochemistry, Molecular Biology and Biophysics, University of Minnesota, 7-194 MCB Building, 420 Washington Ave SE, Minneapolis, MN 55455 USA; 2 Department of Medicinal Chemistry and Molecular Pharmacology, College of Pharmacy, Purdue Center for Cancer Research, Purdue University, West Lafayette, IN 47907 USA

## Abstract

Fluorescent drug screening assays are essential for tyrosine kinase inhibitor discovery. Here we demonstrate a flexible, antibody-free TR-LRET kinase assay strategy that is enabled by the combination of streptavidin-coated quantum dot (QD) acceptors and biotinylated, Tb^3+^ sensitizing peptide donors. By exploiting the spectral features of Tb^3+^ and QD, and the high binding affinity of the streptavidin-biotin interaction, we achieved multiplexed detection of kinase activity in a modular fashion without requiring additional covalent labeling of each peptide substrate. This strategy is compatible with high-throughput screening, and should be adaptable to the rapidly changing workflows and targets involved in kinase inhibitor discovery.

Protein tyrosine kinases have been significant drug targets for decades, and an ever-growing number of compounds are being tested against various kinases for their therapeutic potential. Fluorescent kinase assays have been the most popular form of kinase inhibitor screening assay in drug discovery practices, implemented by a variety of strategies^1^,^2^,^3^. Many fluorescent kinase assays use time-resolved fluorescence/luminescence (TRF/TRL) and/or fluorescence/luminescence resonance energy transfer (FRET/LRET)^1^,^4^,^5^. One universal feature shared by these assays is their high dependency on customized reagents, notably the requirement for specialized antibodies labeled by lanthanide chelates and their derivatives^1^,^2^. These labeled antibodies are usually paired with substrates/secondary antibodies that are labeled with organic fluorophores, so that the requirements of LRET-based detection for donor and acceptor fluorophores are satisfied^1^,^6^. While many of the current popular LRET assay kits were designed based on this strategy, the dependency on customized antibody conjugates has resulted in high associated costs, laborious handling requirements, and can be limited by antibody availability for a given target’s substrate(s). Small organic fluorophores can be used for TR-LRET, but also face limitations to higher order multiplexing such as small dynamic range, small Stokes shifts, and spectral bleed through, affecting signal to noise and sensitivity. Although post-experiment *in silico* correction is possible in combination with customized instruments, the amount of extra work and cost could be significantly amplified when screening large compound libraries^7^,^8^. For these reasons, new TR-LRET detection strategies that offer antibody-free multiplexed monitoring, increased convenience, and better cost efficiency would be helpful tools to the ongoing drug discovery efforts on various kinase targets.

Quantum dots (QD) have many advantages over conventional organic fluorophores, and have been intensively investigated as a potential platform for a variety of biosensing applications^9^,^10^,^11^, including kinase assays and high-throughput screening^12^,^13^,^14^,^15^,^16^. As nanosized semiconductor fluorophores, QDs have high quantum yield, size-dependent emission spectra, and resistance to photobleaching^17^,^18^. Various surface modification options are also available to QD, enabling their functionalization and application in a wide range of chemical biology applications. Previous studies have employed many different strategies to establish QD-based kinase assays, such as charge-dependent detection^15^, antibody-based FRET detection^12^,^14^, antibody-based quenching detection^13^, or FRET detection facilitated by labeled ATP^16^. While such methods demonstrate the advantage of using QDs as either donor or acceptor in FRET/LRET assays, most still rely on antibodies and/or chemical labeling with lanthanide chelates^12^,^14^,^19^ or depend on mechanisms that would not allow multiple reactions in one well^15^,^16^.

We previously reported phosphorylation-sensitive lanthanide binding peptides as specialized substrates for tyrosine kinases^20^. As also reported by others^21^,^22^, these substrates chelate lanthanide ions directly upon phosphorylation, eliminating the need for chemical labeling with a separate lanthanide chelate^22^,^23^,^24^, resulting in higher lanthanide luminescence intensity and longer luminescence lifetime^20^,^21^,^23^. The *in silico* workflows developed in our lab have ensured the optimal kinase specificity as well as lanthanide binding affinity simultaneously for biosensors that are newly designed^25^ or engineered from existing substrates^26^, providing the foundation of multiplexed kinase assay. We have explored the design and application of such sequences^25^ for novel time-resolved luminescence kinase assays in TRL and TR-LRET forms^20^,^27^ for a variety of kinases involved in cancer signaling, including a dual-plexed approach using small molecule fluorophores to differentiate between substrates^27^. While our previous approach is functional and high-throughput compatible, its modularity was not optimal—requiring covalent fluorophore labeling and purification of each individual peptide substrate. Here we report a more flexible strategy for a multiplexed, antibody-free kinase assay using TR-LRET between quantum dot (QD) fluorophores and phosphorylation-dependent lanthanide-sensitizing peptide biosensors. Because of the broad and continuous absorption spectra of QDs, which are highest in the UV to short wavelength visible range regardless of emission color (Fig. 1a), the luminescence emission from Tb^3+^ is efficiently exploited and provides more flexible LRET pair options (Fig. 1a) than conventional organic fluorophores.

## Results and Discussion

**Figure 1:**
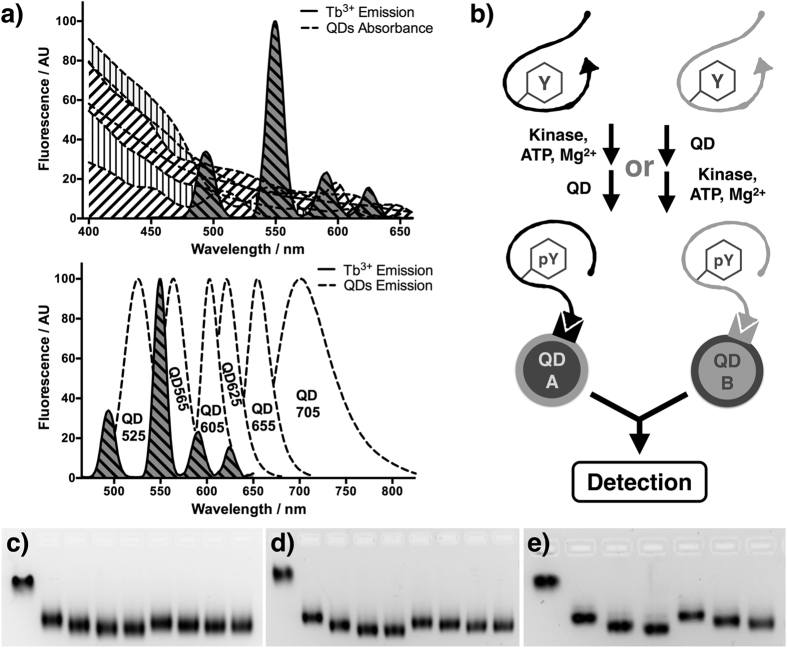
Rationale for using streptavidin-coated QD and Tb^3+^ sensitizing biosensor to establish time-resolved LRET kinase assay. (**a**) Top panel: spectral overlap of Tb^3+^ emission spectrum and various QD absorption spectra. Bottom panel: multi-color detection which could potentially be enabled by tunable QD emission spectra. (**b**) General workflow of multiplexed tyrosine kinase assay using QD-biosensor conjugates. The conjugates can be prepared either before or after the kinase assay for multi-color time-resolved LRET detection. Triangle key-lock indicates biotin-streptavidin binding. (**c**–**e**) The formation of QD-biosensor conjugates used in this study was evaluated by electrophoresis on 1% agarose gel. (**c**) QD605-SAStide conjugate. Lanes (left to right): QD/pSAStide ratio 1:0, 1:50, 1:100, 1:200, 1:300, QD/SAStide ratio 1:50, 1:100, 1:200, 1:300. (**d**) QD655-SFAStide-A conjugate. Lanes (left to right): QD/pSFAStide-A ratio 1:0, 1:50, 1:100, 1:200, 1:300, QD/SFAStide-A ratio 1:50, 1:100, 1:200, 1:300. (**e**) QD655-SFAS-A conjugate with the presence of 2.4 M urea (the quenching reagent used in kinase assay). Lanes (left to right): QD/SFAStide-A (without urea) ratio 1:0, 1:50, 1:100, 1:200, QD/SFAStide-A (with urea) ratio 1:50, 1:100, 1:200.

As depicted in Fig. 1b, QD-peptide conjugates could be readily prepared via the interaction between tetravalent streptavidin and biotin. Streptavidin-coated^28^ QD605 or QD655 were incubated with SAStide (GGDEEDYEEPDEPGGK_b_GG, a Syk biosensor^25^) or SFAStide-A (GGEEDEDIYEELDEPGGK_b_GG, a Src family kinase biosensor^25^) at room temperature in HEPES buffer (pH = 7.5) for 1 hr. To confirm the formation of the conjugates, agarose gel electrophoresis^14^ was performed (Fig. 1c,d). QD-biosensor conjugates exhibited increased electrophoretic mobility relative to unlabeled QDs. QDs were saturated (exhibiting no further electrophoretic mobility changes) when biosensor/QD ratio reached about 200:1. Conjugate formation was resistant to high concentrations of urea (Fig. 1e), whether the urea addition was prior to or after the incubation process. The QD-biosensor conjugates were also subject to ligand exchange tests, in which the pre-made conjugates showed no signal loss caused by ligand exchange with another labeled QD or excess amount of free biosensors in buffer during a 4 hr incubation (Figures S12 and S13), indicating their high stability (deriving from the high affinity of the streptavidin-biotin interaction).

**Figure 2:**
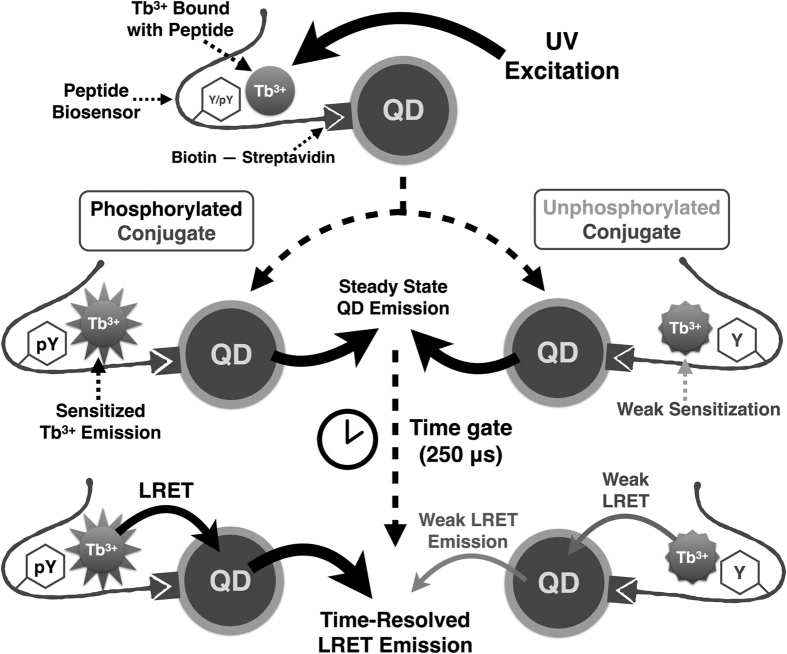
Schematic mechanism of time-resolved LRET detection using phosphorylated QD-biosensor conjugate. Phosphorylated biosensor leads to stronger time-resolved LRET emission from the conjugated QD, whereas unphosphorylated biosensor leads to weak LRET emission. Actual conjugates have multiple biosensors bound to QD surface.

**Figure 3:**
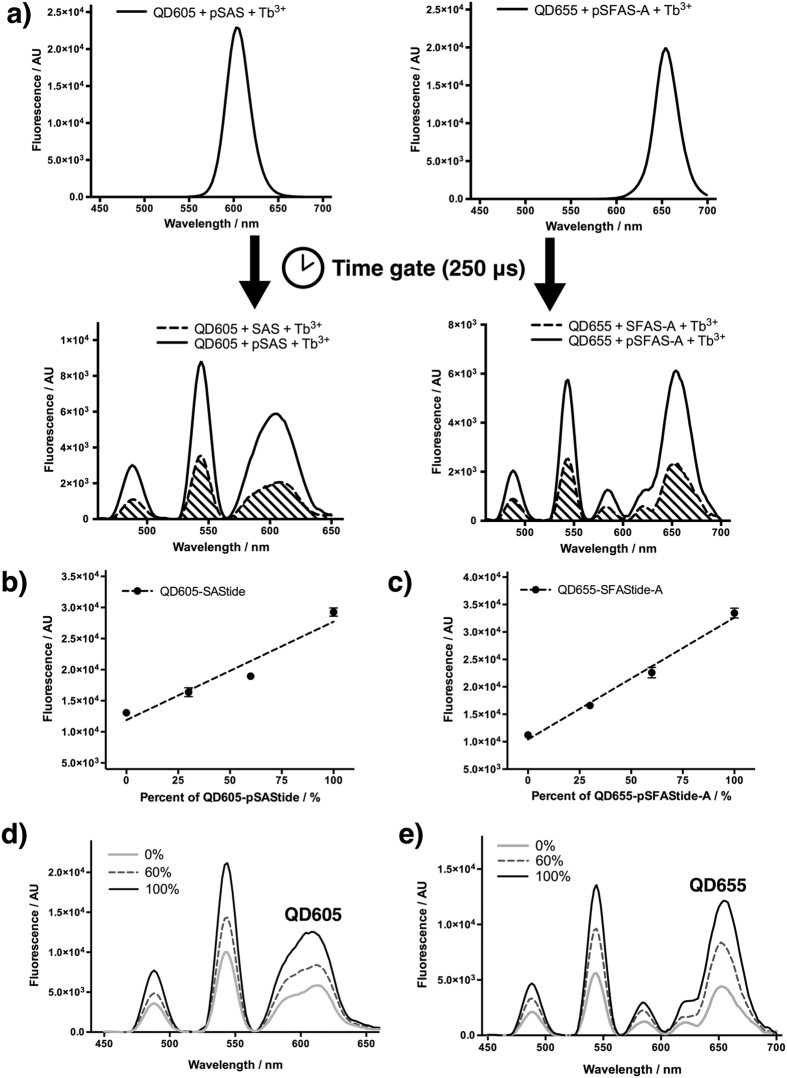
Time-resolved LRET emission of QD-biosensor conjugates. (**a**) Emission spectra comparing steady-state and time-resolved emission of the QD-biosensor conjugates used in this study. Steady state emission from QD605-pSAStide (10 nM) and QD655-pSFAStide-A (10 nM) conjugate are dominated by inherent QD emission. However, time-resolved LRET emission from both conjugates showed a significant difference between emission spectra of the phospho- and unphospho-conjugates. (**b**) In order to mimic the experimental conditions of kinase assay (as discussed in Figure S11), a calibration curve for QD605-SAStide was established in kinase reaction buffer. (**c**) In order to mimic the experimental condition of multiplexed kinase assay (as discussed in Figure S11), a calibration curve for QD655-SFAStide-A was established in kinase reaction buffer. (**d**) Emission spectra of samples from b) showed that TR-LRET emission increased as the percent of pSAStide (proportional to total biosensor) increased. (**e**) Emission spectra of samples from (**c**) showed that TR-LRET emission increased as the percent of pSFAStide-A (proportional to total biosensor) increased. For (**b**,**c**) the QD-biosensor conjugates were diluted to 20 nM with 100 μM Tb^3+^ in 10 mM HEPES buffer (pH = 7.5) and other kinase reaction reagents as described in Supporting Information. Time-resolved emission spectra were recorded using built-in monochromator on a Synergy 4 microplate reader with 250 μs delay and 1000 μs integration. Excitation wavelength: 266 nm. Calibration curves were established using luminescence reading from a 605/10 and a 655/10 filter accordingly with a 265/10 excitation filter.

The peptide biosensors were designed to chelate free Tb^3+^ cation when phosphorylated by tyrosine kinases as mentioned above and described in our previous publications^20^,^25^,^26^. Once the conjugates were prepared, luminescence emission was generated upon UV excitation, from both the QD (short-lived fluorescence) and the peptide-chelated Tb^3+^ (long-lived luminescence) (Fig. 2). Indeed, steady state emission of the conjugates was dominated by inherent QD fluorescence regardless of peptide phosphorylation state as expected (Fig. 3a), given the much higher extinction coefficients of QDs^28^ (4.4 × 10^6^ cm^−1^ M^−1^ for QD605 at 350 nm and even higher for QD655) than that of Tb^3+^ chelates^29^ (~1.2 × 10^4^ cm^−1^ M^−1^ at 327 nm). However, since QD fluorescence lifetimes are typically less than a hundred nanoseconds, QD inherent emission was greatly reduced by applying a sufficient time gate between excitation and detection (50 μs or longer), while the luminescence emission from Tb^3+^ remained strong. Since Tb^3+^ bound to phosphorylated biosensors had higher luminescence intensity and longer lifetime^20^ than the unphosphorylated conjugate, phosphorylation of QD-biosensor conjugates resulted in stronger LRET emission from the QDs (Figs 3a, S10 and S12). Background QD emission in unphosphorylated conjugate samples was low, and was a combined result of residual QD steady-state fluorescence^30^, free Tb^3+^ binding by buffer components, and unphosphorylated biosensor, which had far lower emission than for the phosphorylated biosensor (as previously described^20^,^25^,^26^ and observed here—arising from lower affinity for Tb^3+^ cation and quenching of Tb^3+^ luminescence by the water molecules that occupy the leftover ligand sites in the unphosphorylated chelate) (Figures S11 and S12). Such background emission could be alleviated by optimizing the reaction buffer components/concentrations and instrumental parameters for sufficient delay times. At 10 nM concentration in the reaction buffer (to mimic multiplexed kinase reaction conditions as discussed in Figure S11), the signal/background for LRET emission from phosphorylated vs. unphosphorylated conjugates was 3:1 when delay time was 250 μs, and could be even higher with longer delay time or higher concentration (Figure S15). Time-resolved emission of both QD605-SAStide and QD655-SFAStide-A conjugates (prepared at biosensor: QD ratio of 100:1 to ensure saturation of the QD surface) showed linear increase with increasing proportion of phosphorylated conjugates, demonstrating that kinase assays using QD-biosensor conjugates can be appropriately quantified according to particular assay conditions and components (Fig. 3b,e). The proportion of phosphorylated conjugate was quantified by either the area under curve (AUC) of QD emission peak spectra or by using readings from designated emission filters, and calibration curves were constructed for quantitative detection of the amount of phosphorylation (Fig. 3b,c). Even for proportions of phosphorylated product of as low as 10% (considered the appropriate regime for kinetics experiments), the Z′ factors calculated for both conjugate (Tables S1–S3) were >0.5, above the threshold that is generally considered the compatibility requirement for high-throughput screening.

**Figure 4:**
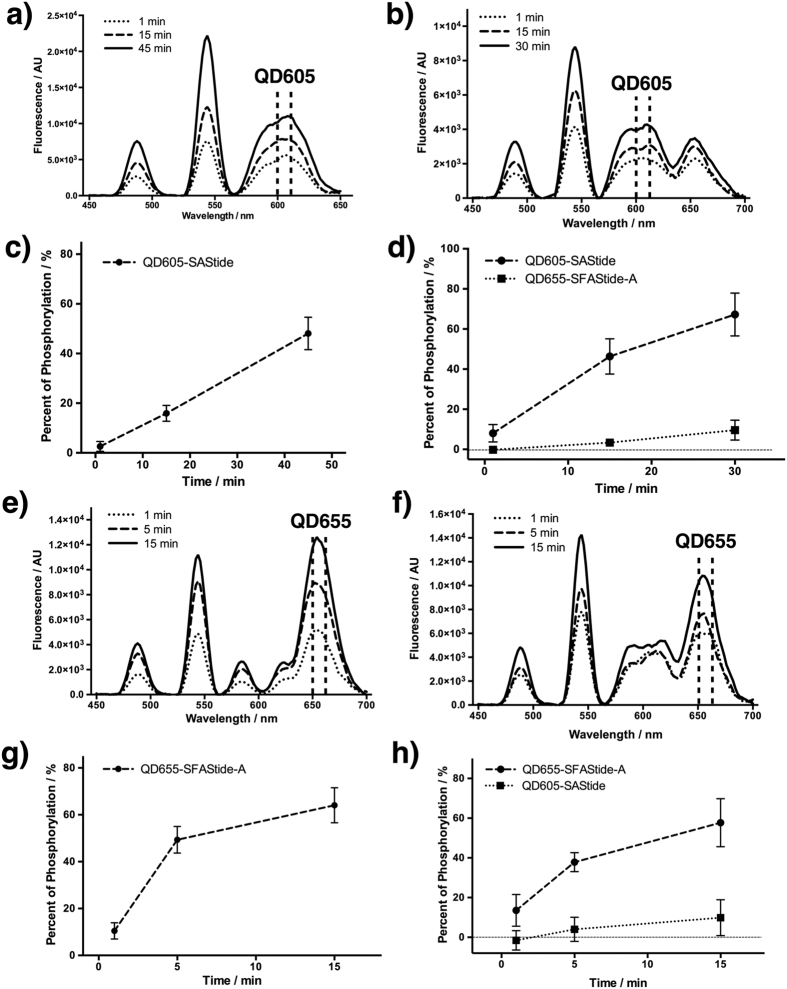
Multiplexed kinase assay demonstrated by using two different QD-biosensor conjugates (final concentration: 20 nM). In general, kinase assays were performed with 15 nM kinase, 5 μM peptides, 50 nM QD, 100 μM ATP, 10 mM Mg^2+^, and 0.2 μg/μL BSA in 25 mM HEPES buffer. Aliquots of samples were quenched in detection buffer (40 μL 6 M urea, 10 μL 1 M NaCl, 10 μL 1 mM Tb^3+^) as detailed in supporting information. TR-LRET emission was quantified by corresponding calibration curves (Figs 3b,c and S16). (**a)** Time-resolved spectra of QD605-SAStide conjugate from Syk kinase assay at 1 min, 15 min, and 45 min after addition of Syk. Conjugate was formed after kinase assay, and 1 μM Na_3_VO_4_ was also included. (**b**) Time-resolved spectra of pre-formed QD605-SAStide and QD655-SFAStide-A conjugates from Syk kinase assay at 1 min, 15 min, and 30 min after addition of Syk. Delay time was set to 400 μs to minimize cross-interference between conjugates. (**c**) Quantification of QD605-SAStide phosphorylation from a) at 1 min, 15 min, and 45 min after addition of Syk. (**d**) Quantification of QD605-SAStide and QD655-SFAStide-A phosphorylation by Syk from (**b**) at  1 min, 15 min, and 30 min after addition of Syk. Specific QD605-SAStide phosphorylation by Syk is shown. Horizontal dashed line, 0% phosphorylation. Dashed line, QD605-SAStide signal. Dotted line, QD655-SFAStide-A signal. (**e**) Time-resolved spectra of QD655-SFAStide-A conjugate from Src kinase assay at 1 min, 5 min, and 15 min after addition of Src. Conjugates were formed before kinase assay. (**f**) Time-resolved spectra of pre-formed QD605-SAStide and QD655-SFAStide-A conjugates from Src kinase assay at 1 min, 5 min, and 15 min after addition of Src. (**g**) Quantification of QD655-SFAStide-A phosphorylation from e) at 1 min, 5 min, and 15 min after addition of Src. (**h**) Quantification of QD605-SAStide and QD655-SFAStide-A phosphorylation by Src from (**f**) at 1 min, 5 min, and 15 min after addition of Src. Specific QD655-SFAStide-A phosphorylation by Src is shown. Horizontal dashed line, 0% phosphorylation. Dashed line, QD655-SFAStide-A signal. Dotted line, QD605-SAStide signal.

An advantage of the QD-biosensor conjugates shown here is that the TR-LRET kinase assay could be set up with flexible protocols in which the conjugates were prepared either prior to or after the kinase reaction. *In vitro* Src and Syk activity assays were performed according to the procedure described in Supporting Information. Briefly, 20 nM of substrate, as either pre-made QD-biosensor conjugates or free biosensor peptide, was incubated with the reaction buffer for 5 min, and the reaction was started by addition of kinase(s). At selected time points, aliquots of the reaction mixture were taken out and quenched in Tb^3+^-containing detection buffer to a final volume of 100 μL. Time-resolved, quantitative readout was done by either monochromator-based spectral scan or filter-based detection using a Biotek Synergy4 plate reader. In the single conjugate kinase assay, TR-LRET analysis of each conjugate displayed a corresponding increase in QD emission signal as a result of biosensor phosphorylation (Fig. 4a,c,e,g). Kinase activity was readily reported by both the conjugate formed prior to the reaction (Fig. 4e,g) and after the reaction (when the quenched aliquot was incubated with QD to capture biotinylated peptide) (Fig. 4a,c), showing that a QD conjugate strategy could be easily adapted to rapidly changing assay needs without prior chemical preparations such as covalent fluorophore labeling.

For multiplexed detection, QD605-SAStide and QD655-SFAStide-A conjugates were both prepared prior to the assay and then combined in reaction buffer before kinases were added (Fig. 4b,d,f,h). TR-LRET spectra for the conjugate mixture were collected by the same method above. To minimize the cross interference caused by the overlap of QD605 background emission with QD655 absorption, delay time for QD605-SAStide detection was increased to 400 μs. In the presence of either Src or Syk, both conjugates showed increasing LRET emission corresponding to the specific respective kinase (Fig. 4b,d,f,h) used over the course of the reactions. Selective phosphorylation of QD605-SAStide by Syk (Fig. 4b,d) and selective phosphorylation of QD655-SFAStide-A by Src (Fig. 4f,h) were demonstrated in the mixture of both conjugates, and the result was in agreement with our previously published work on these two substrates^20^,^25^. TR-LRET emission can be quantified in percent of phosphorylation for either single conjugate assay (Fig. 4c,g) or multiplexed assay (Fig. 4d,h) using corresponding calibration curves (Figs 3b,c and S16). These results demonstrated the feasibility of using QD-biosensor conjugates in multiplexed TR-LRET kinase assays with a robust readout and flexible procedures.

In conclusion, this study has implemented QDs as the LRET acceptor in a time-resolved LRET kinase assay strategy that uses phosphorylation-sensitive Tb^3+^-chelating synthetic peptides, demonstrated with tyrosine kinases Src and Syk. Previous studies have suggested that QDs are not ideal LRET acceptors in steady state detection^31^, indicating the importance of using time-resolved detection for the assays discussed in this study. The major advantage of this strategy is that mainstream TR-LRET assays used in kinase inhibitor screening practices today are highly dependent on chemically-labeled, customized antibodies^1^,^5^, which are high-cost and require significant, and unique, investment to implement per target. By using validated lanthanide binding peptide biosensors for the kinases of interest^25^, which are inexpensive to produce and do not require any labeling other than standard incorporation of biotinyl-Lysine during their synthesis, the strategy reported here enables flexible combinations of QD/biosensor LRET pairs for assaying a broad, expandable list of kinases^25^ in a cost efficient manner. Additionally, this approach is more modular than our previously reported dual-plexed LRET assay^27^, since the choice of LRET donor and acceptor can be decided immediately before the assay without any special chemical labeling planned ahead. The simple nature of this antibody-free assay also renders it more adaptable to the rapidly changing needs of new assay targets, and thus it could be a valuable tool for drug discovery efforts.

## Methods and Materials

### Peptide synthesis

Peptides were prepared to >90% purity using standard Fmoc solid phase peptide synthesis procedure as described in the Supporting Information. Briefly, peptides were synthesized using a Symphony X peptide synthesizer (Protein Technologies, USA) on Rink-amide-MBHA resin (Protein Technologies, USA). The synthesis was performed using Fmoc (9-fluorenylmethoxy-carbonyl)-protected amino acids (Protein Technologies, USA) with coupling reagent 2-(6-chloro-1H-benzotria-zole-1-yl)-1,1,3,3-tetramethylaminiumhexafluorophosphate (HCTU, Protein Technologies, USA) in the presence of N-methylmorpholine (NMM, Protein Technologies, USA) in dimethylformamide (DMF, Iris Biotech Gmbh, Germany). A 20% piperidine solution in DMF was used for Fmoc deprotection. Peptide cleavage and side-chain deprotection were performed simultaneously by using 10 mL mixture of trifluoroacetic acid (TFA, Protein Technologies, USA)/water/ethane dithiol (EDT, Sigma-Aldrich, USA)/triisopropylsilane (TIS, Sigma-Aldrich, USA) (94:2.5:2.5:1, v/v). Cleaved peptides were then precipitated and washed three times by cold diethyl ether (Fisher Scientific, USA). Precipitated peptides were redissolved and lyophilized, followed by LC-MS characterization, and HPLC purification.

### Gel electrophoresis

Streptavidin-coated QD605ITK or QD655ITK (2 μM stock solution, Thermo Fisher, USA) and peptides were diluted into 10 mM 2-[4-(2-hydroxyethyl)-piperazin-1-yl]ethanesulfonic acid (HEPES, Calbiochem, USA) buffer (pH = 7.5). The final solutions had 10 nM QDs, various amount of peptides, and 2.4 M urea when indicated. After incubation for 1 hr at room temperature, glycerol (100%, Macron Fine Chemicals, USA) was added to each sample with a final concentration of 5% (v/v). The QD-biosensor conjugates were then loaded to a 10 cm long 1% (w/v) agarose (Invitrogen, USA) gels (4 μL sample per well) in 1 × TAE buffer (Thermo Fisher Scientific, USA), and run for 60 min at 100 V using BioRad PowerPac Basic power supply. Gels were then imaged using a Gel Logic 112 imaging system (Carestream, USA).

### Fluorescence/luminescence measurements

Fluorescence/luminescence emission spectra were measured on a Synergy4 plate reader (Biotek, USA) at room temperature in 384-well black plates (Fluortrac 200, Greiner bio-one, Germany). The emission spectra were collected between 450 and 650 nm with 2 nm increments using the built-in monochromator. Filter-based detection using 550/10, 605/10, 655/10 emission filters (Omega Optical, USA) was also applied when necessary. The excitation wavelength was set to 266 nm by built-in monochromator, or a 265/10 excitation filter (Omega Optical, USA) with 250 μs delay time unless otherwise indicated. Details are described in the Supporting Information.

### Kinase assay

QDs were incubated with the biosensors in HEPES buffer for 1 hr to form the conjugates. Alternatively, QDs could also be added to the quenched samples after the kinase assay. Final kinase reaction buffer (e.g. 15 nM Src, 5 μM peptides, 100 μM adenosine triphosphate (ATP), 10 mM MgCl_2_, 0.2 μg/μL BSA, 25 mM HEPES, pH 7.5) were incubated at 37 °C, and the reaction was initiated by the addition of kinase. At selected time points, 40 μL aliquots were taken and quenched in detection buffer (40 μL 6 M urea, 10 μL 1 mM TbCl_3_ and 10 μL 1 M NaCl). Time-resolved luminescence emission spectra were collected as described in Supporting Information.

## Additional Information

**How to cite this article**: Cui, W. and Parker, L. L. Modular, Antibody-free Time-Resolved LRET Kinase Assay Enabled by Quantum Dots and Tb^3+^-sensitizing Peptides. *Sci. Rep.* **6**, 28971; doi: 10.1038/srep28971 (2016).

## Supplementary Material

Supplementary Information
