# Supplementary material for: Modular, Antibody-free Time-Resolved LRET Kinase Assay Enabled by Quantum Dots and Tb3+-sensitizing Peptides
Source: Sci Rep. 2016 Jul 18;6:28971. doi: 10.1038/srep28971 (PMC4947905; doi:10.1038/srep28971)
Supplement: Supplementary Information [file srep28971-s1.pdf]

Supporting Information

Modular, Antibody-free Time-Resolved LRET Kinase Assay Enabled by Quantum Dot and Tb^{3+}-sensitizing Peptides

*Wei Cui, and Laurie L. Parker**

Peptide synthesis: Peptides were synthesized using a Symphony X peptide synthesizer (Protein Technologies, USA) on Rink-amide-MBHA resin (Protein Technologies, USA). The peptides were synthesized at a 50 μmol scale. The synthesis was performed using Fmoc (9-fluorenylmethoxy-carbonyl)-protected amino acids (Protein Technologies, USA) with coupling reagent 2-(6-chloro-1H-benzotria- zole-1-yl)-1,1,3,3-tetramethylaminium hexafluorophosphate (HCTU, Protein Technologies, USA) in the presence of N-methylmorpholine (NMM, Protein Technologies, USA) in dimethylformamide (DMF, Iris Biotech Gmbh, Germany). A 20% piperidine solution in DMF was used for Fmoc deprotection. Peptide cleavage and side-chain deprotection were performed simultaneously by using 10 mL mixture of trifluoroacetic acid (TFA, Protein Technologies, USA)/water/ethane dithiol (EDT, Sigma-Aldrich, USA)/triisopropylsilane (TIS, Sigma-Aldrich, USA) (94:2.5:2.5:1, v/v). Cleaved peptides were then precipitated and washed three times by cold diethyl ether (Fisher Scientific, USA). Precipitated peptides were re-dissolved in acetonitrile/water/TFA (50:50:0.1, v/v), flash frozen by liquid nitrogen, and lyophilized.

Peptide purification: The lyophilized peptides were verified by a customized liquid chromatography-mass spectrometry (LC-MS) system based on Agilent 1200 series LC and Agilent 6130 quadrupole ESI-MS. Peptides were purified on a Hewlett-Packard 1100 Series preparative reverse-phase high performance liquid chromatography (HPLC, Hewlett-Packard, USA) equipped with a Sepax Bio-C18 reverse-phase column (Sepax Technologies, USA).

Purified peptides were verified again on the same LC-MS system described above. The chromatographic characterization of peptides used in this study was shown as below.

Blank run on the customized LC-MS system:

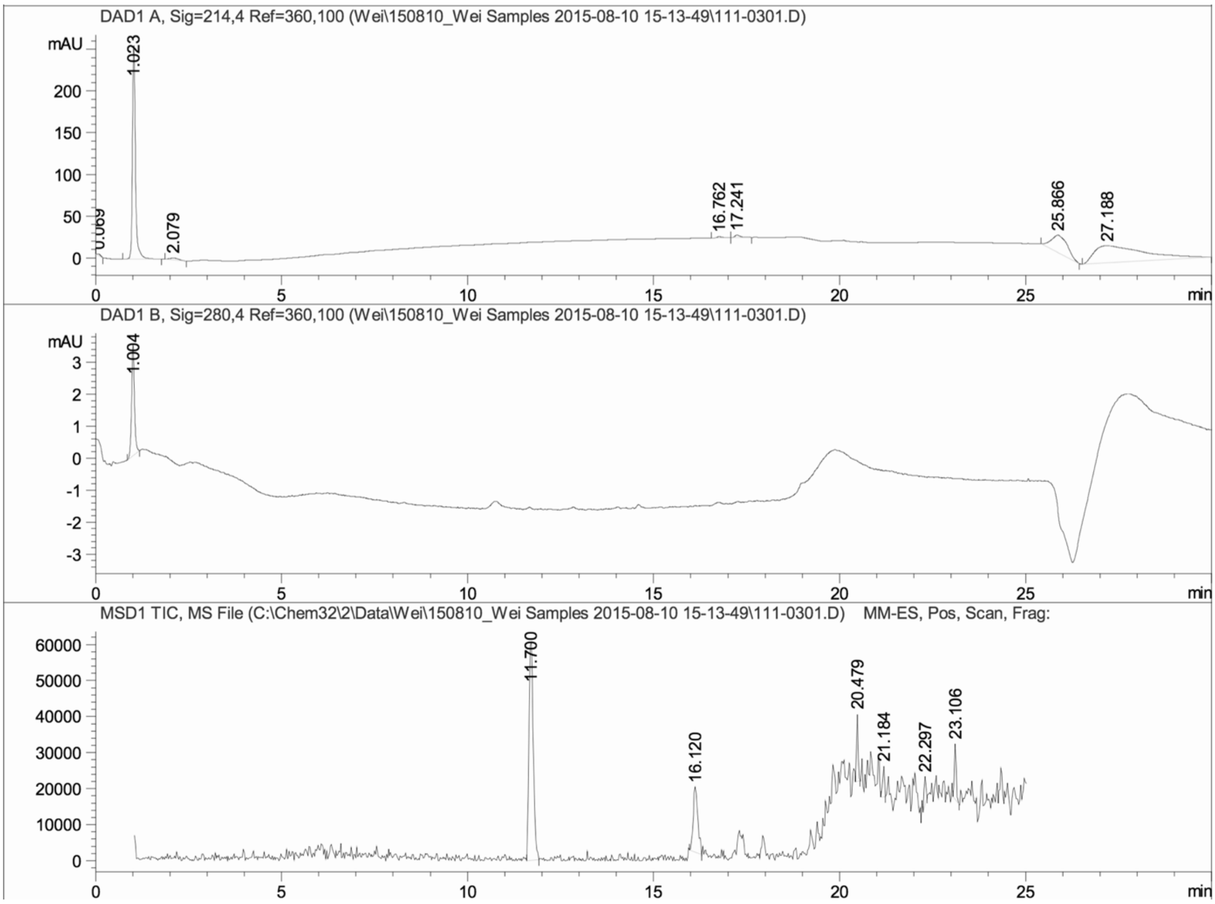

Figure S1. Blank sample run on the LC-MS system. Notice the background TIC peak at t = 11.7 min and t = 16.1 min.

LC-MS analysis of SAStide (Sequence: GGDEEDYEEPDEPGGK$_b$GG, K$_b$ = biotinylated lysine):

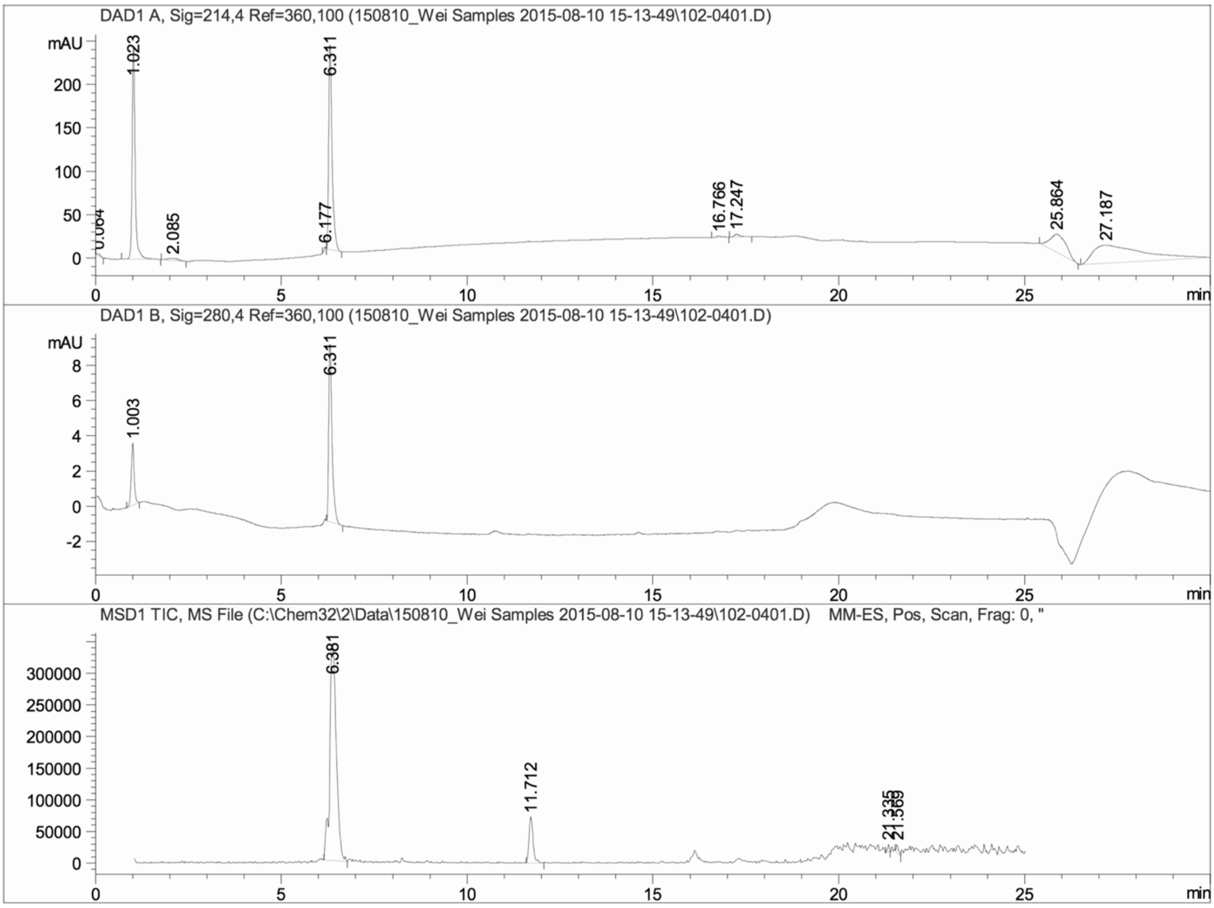

Figure S2. LC-MS analysis of SAStide, retention time t = 6.38 min.

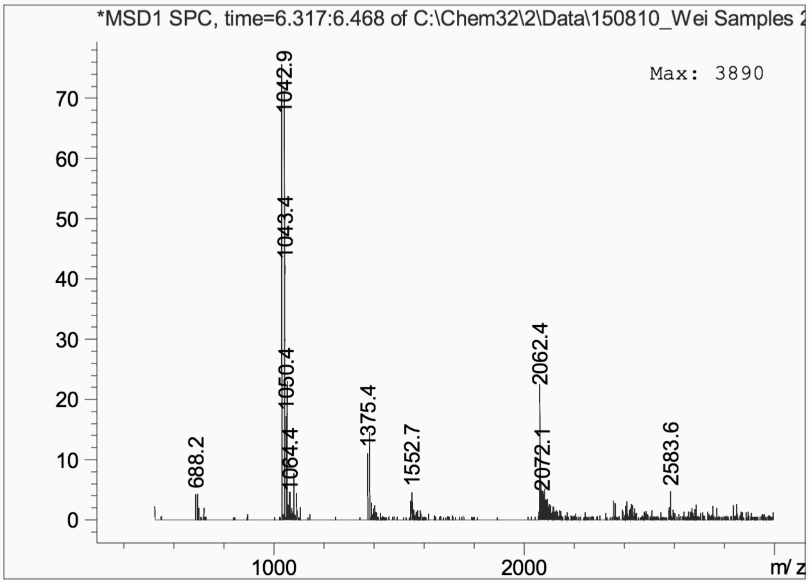

Figure S3. Mass spectrum of the peptide peak at t = 6.38 min.

LC-MS analysis of pSAStide (Sequence: GGDEEDpYEEPDEPGGK_bGG, pY = phosphorylated tyrosine, K_b = biotinylated lysine):

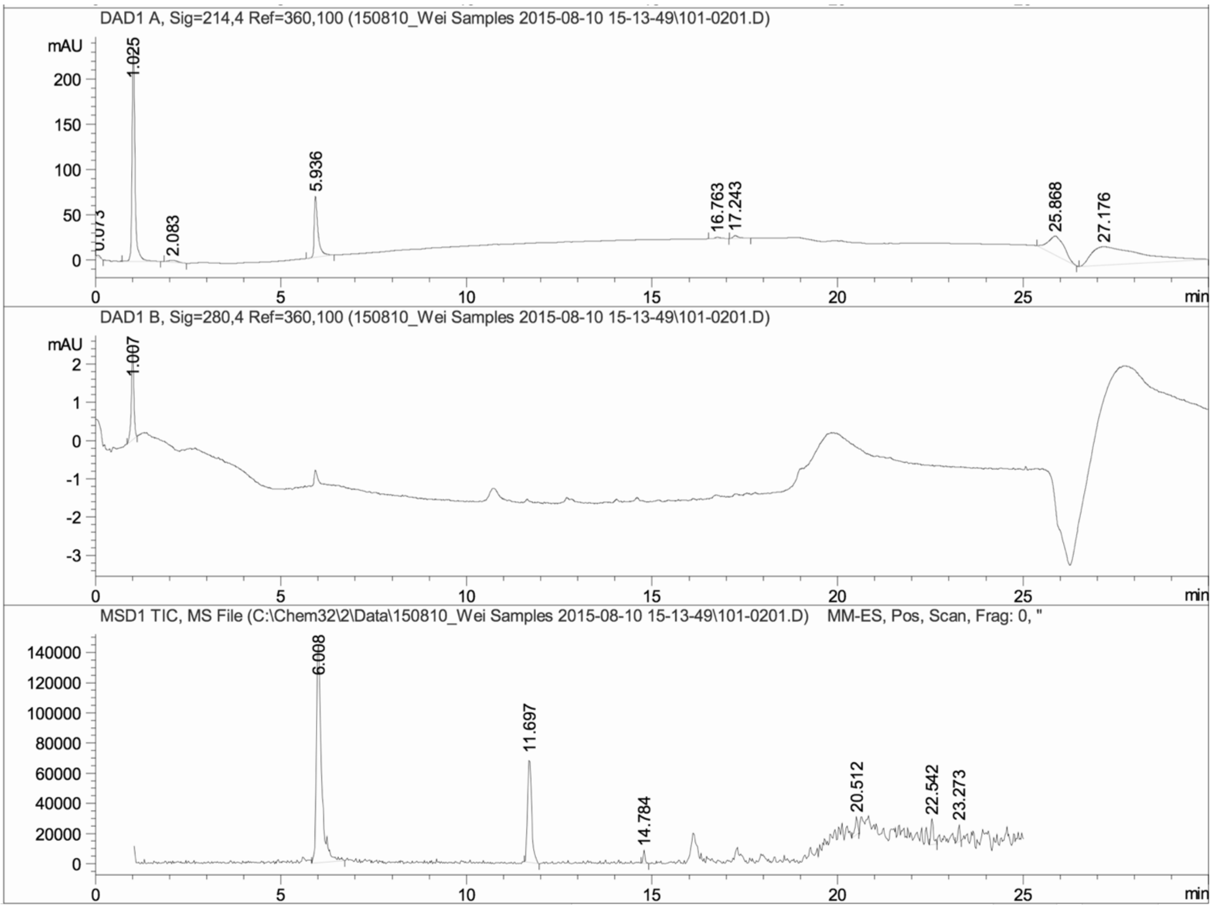

Figure S4. LC-MS analysis of pSAStide, retention time t = 6.01 min.

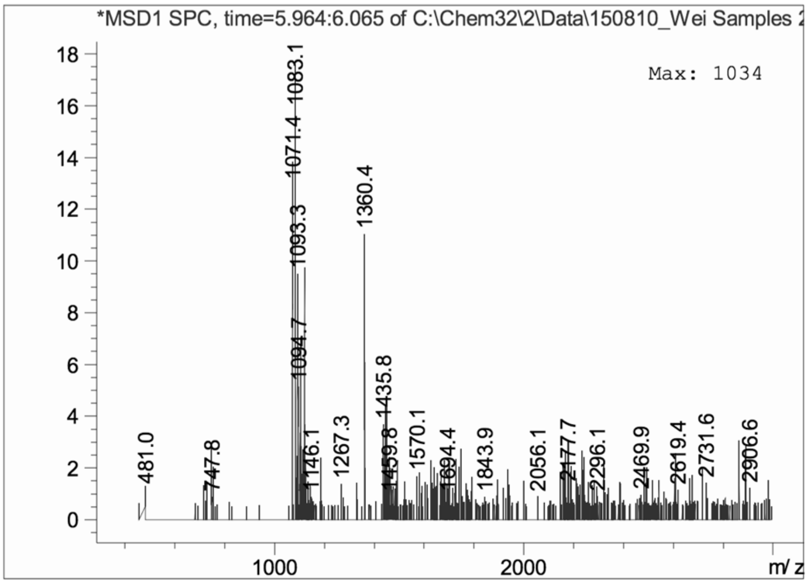

Figure S5. Mass spectrum of the peak at t = 6.01 min.

LC-MS analysis of SFAStide-A (Sequence: GGEEDEDIYEELDEPGGK$_b$GG, K$_b$ = biotinylated lysine):

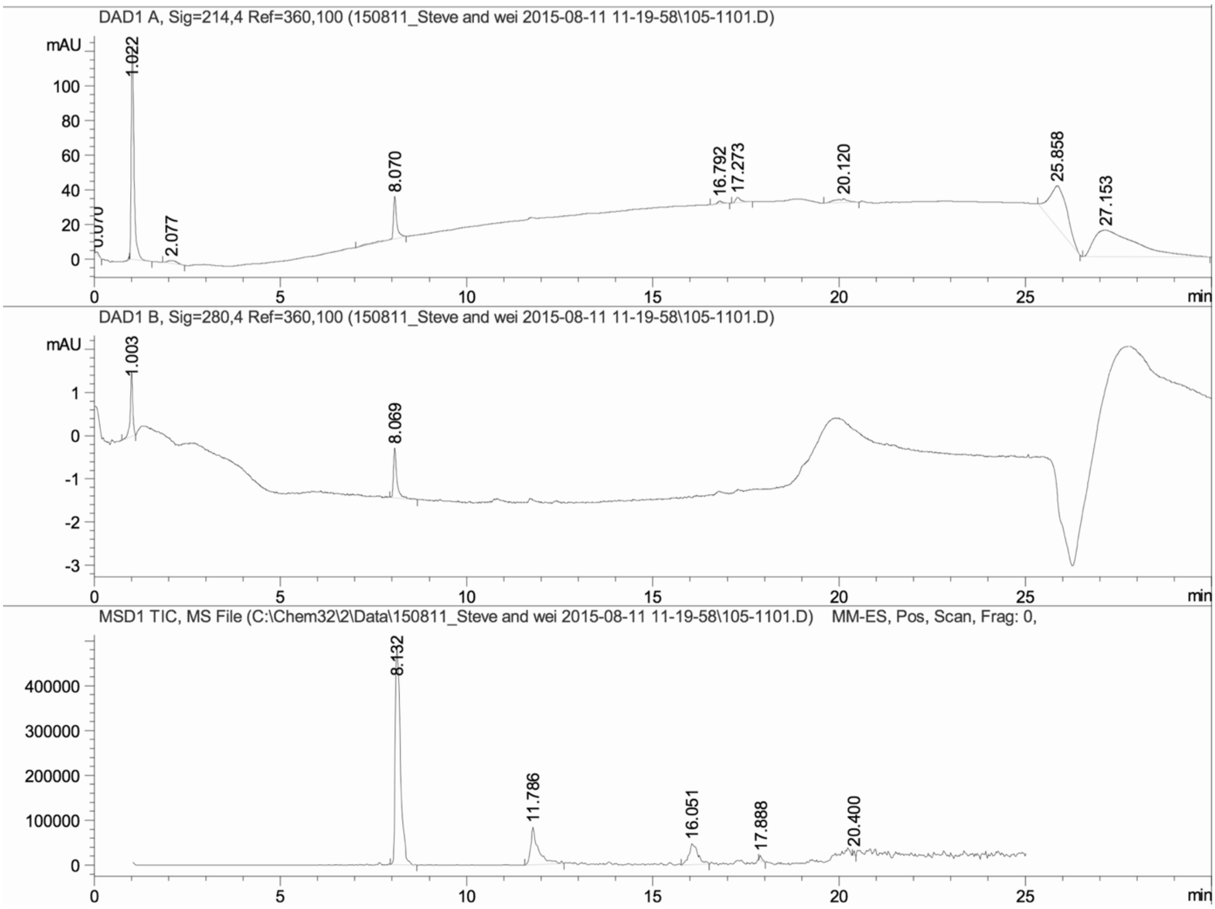

Figure S6. LC-MS analysis of SFAStide-A, retention time t = 8.13 min.

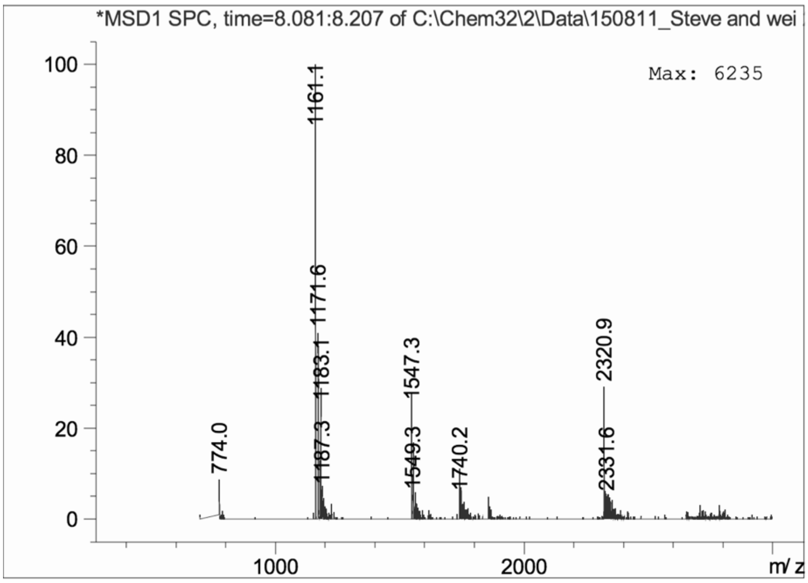

Figure S7. Mass spectrum of the peak at t = 8.13 min.

LC-MS analysis of pSFAStide-A (Sequence: GGEEDEDIpYEELDEPGGK$_b$GG, pY = phosphorylated tyrosine, K$_b$ = biotinylated lysine):

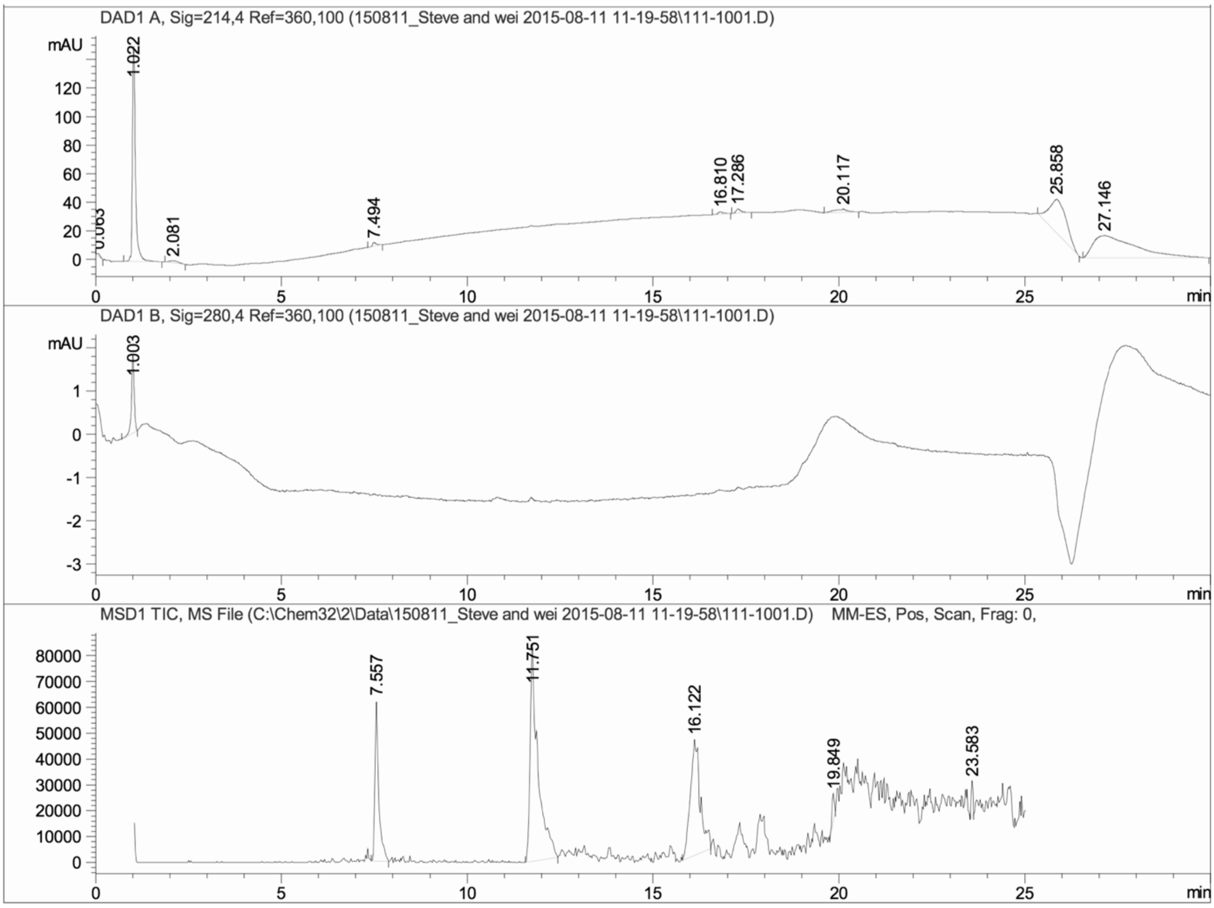

Figure S8. LC-MS analysis of pSFAStide-A, retention time t = 7.56 min.

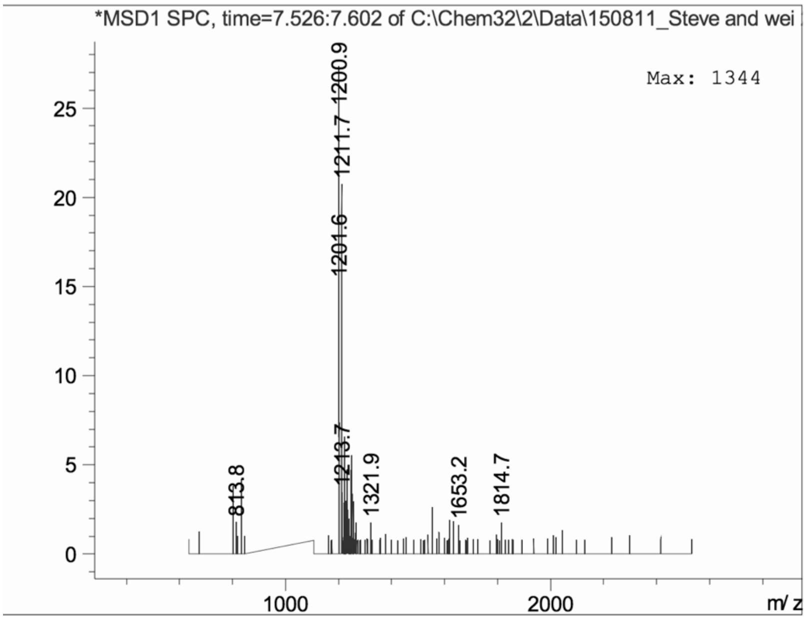

Figure S9. Mass spectrum of the peak at t = 7.56 min.

Gel electrophoresis: Streptavidin-coated QD605ITK or QD655ITK (2 μM stock solution, Thermo Fisher, USA) and peptides were diluted into 10 mM 2-[4-(2-hydroxyethyl)-piperazin-

1-yl]ethanesulfonic acid (HEPES, Calbiochem, USA) buffer (pH = 7.5). The final solutions had 10 nM QDs, various amount of peptides, and 2.4 M urea when indicated. After incubation for 1 hr at room temperature, glycerol (100%, Macron Fine Chemicals, USA) was added to each sample with a final concentration of 5% (v/v). The QD-biosensor conjugates were then loaded to a 10 cm long 1% (w/v) agarose (Invitrogen, USA) gels (4 μL per well) in 1× TAE buffer (Thermo Fisher Scientific, USA), and run for 60 min at 100 V using BioRad PowerPac Basic power supply. Gels were then imaged using a Gel Logic 112 imaging system (Carestream, USA). The QD-biosensor conjugate exhibited increased mobility when compared with unlabeled QDs. The mobility did not change at biosensor/QD ratios higher than 200:1, indicating the saturation of biotin binding sites on the QD surface.

Fluorescence/luminescence measurements: Lanthanide luminescence emission spectra were measured on a Synergy4 plate reader (Biotek, USA) at room temperature in 384-well black plates (Fluortrac 200, Greiner bio-one, Germany). The excitation wavelength was set to 266 nm using the built-in monochromator, or a 265/10 excitation filter (Omega Optical, USA). Time-resolved luminescence spectra were collected between 450 and 650 nm with 2 nm increments using the built-in monochromator, or using 550/10, 605/10, 655/10 emission filters (Omega Optical, USA). Ten technical replicate readings were performed for each well. Delay time was usually 250 μs unless otherwise indicated, and measurement time was 1 ms. All other instrumental parameters (e.g. sensitivity, a parameter of the Synergy4 that is similar to gain) were fixed. Both the spectral area under the curve (AUC) and the intensity reading from emission filters could be used for quantification of luminescence emission.

Kinase assay: Active recombinant Src and Syk were purchased from a commercial manufacturer (Millipore, USA). QDs were incubated with the biosensors in HEPES buffer for 1 hr to form the conjugates, and then other reagents were added. Final kinase reaction buffer

(e.g. 15 nM kinase, 5 μM peptides, 100 μM adenosine triphosphate (ATP), 10 mM $MgCl_2$, 0.2 μg $μL^{-1}$ BSA, 25 mM HEPES, pH 7.5) were incubated at 37 °C, and the reactions were initiated by adding kinases to a final concentration of 15 nM. Alternatively, QDs could also be added to the quenched samples after the kinase assay. At selected time points, 40 μL of aliquots were taken and quenched in detection buffer (40 μL 6 M urea, 10 μL 1 mM $TbCl_3$ and 10 μL 1 M NaCl). The final concentration of QD-biosensor conjugates was 20 nM (1:100 QD:peptide ratio), and that of Tb^{3+} was 100 μM (in a total volume of 100 μL). Time-resolved luminescence emission spectra were collected as described above.

Effect of LRET on Tb^{3+} emission: The energy transfer from Tb^{3+} to QD was clearly demonstrated in Figure S10. When increasing Tb^{3+} concentration for the purpose of assay optimization as described below, both Tb^{3+} emission and LRET emission peaks increased accordingly (Figure S11).

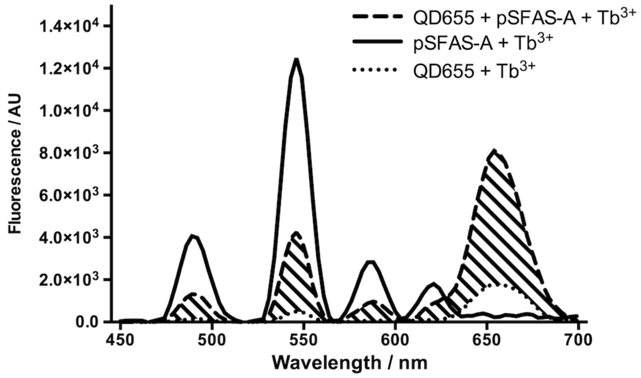

Figure S10. Tb^{3+} emission with or without LRET. QD655-pSFAS-A conjugate was prepared at 1:100 QD:biosensor ratio and final concentration was 10 nM. The concentration of free pSFAS-A was 1 μM, resulting in the same amount of biosensor used in preparation of QD-biosensor conjugate. The concentration of free Tb^{3+} was 10 μM in 10 mM HEPES buffer (pH = 7.5). A significant drop in Tb^{3+} emission (dashed line) as well as increase in LRET emission was observed when comparing with free pSFAS-A-Tb^{3+} chelate (solid line). Background emission from unlabeled QD and free Tb^{3+} was also shown in dotted line.

Effect of reaction buffer components on luminescence signal: The signal to noise ratio of this assay could be affected by various assay components, particularly ATP (Lipchik A.M., Parker L.L.. Anal. Chem., 2013, 85: 2582-8) (**Figure S10**).

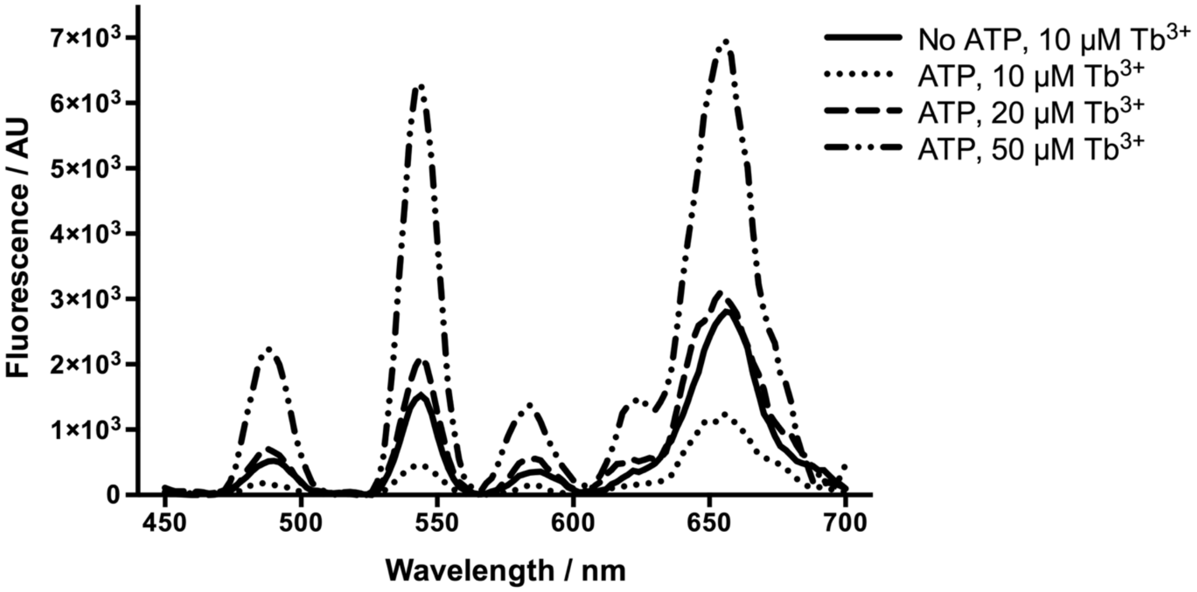

Figure S11. Time-resolved luminescence signal reduction caused by ATP could be reversed by increasing [Tb^{3+}]. The concentration of QD655-pSFAStide-A conjugate was 10 nM and that of ATP was 100 μM.

The three phosphate groups of ATP can act Tb^{3+} ligand and compete with QD-biosensor conjugates for Tb^{3+} binding, especially when the concentration of the conjugate used (5 μM) was 20× lower than that of ATP (100 μM). The introduction of ATP led to approximately 50% decrease in LRET emission signal (Figure S11), but such loss could be reversed by increasing Tb^{3+} concentration in the detection buffer. This method should also be helpful if any other potential Tb^{3+} ligands were introduced due to the requirement of particular assays.

Background LRET emission: Even though a time gate was applied for TR-LRET detection, background emission still existed in detection.

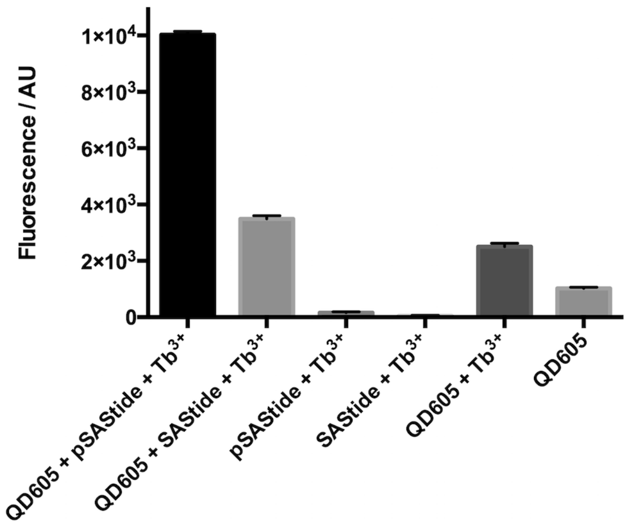

Figure S12. Background time-resolved LRET emission demonstrated by QD605-SAStide conjugates in 10 mM HEPES buffer (pH = 7.5). Detection was done by using a 605/10 emission filter with 200 μs delay time and 1 ms integration time. Conjugate concentration was 10 nM and that of Tb^{3+} was 10 μM.

Free Tb^{3+} could still resulted in additional LRET emission alongside with residual QD steady state emission (**Figure S12**). Such background LRET emission was not caused by minor Tb^{3+} peaks at 580 nm or 620 nm as shown in Figure S12, but more likely to be the result of dynamic interaction of Tb^{3+} with buffer components, such as HEPES or ATP. Unphospho-conjugates also had some LRET emission, due to its lower affinity binding to Tb^{3+}, however it did not compromise the dynamic range required for the TR-LRET assay (as demonstrated by the signal window and Z' factor analyses from the calibration curves).

Ligand exchange of QD-biosensor conjugates: The stability of streptavidin-biotin interaction that maintained the QD-biosensor conjugate was important to future applications in complex biological environment. A ligand exchange assay was performed to examine their stability.

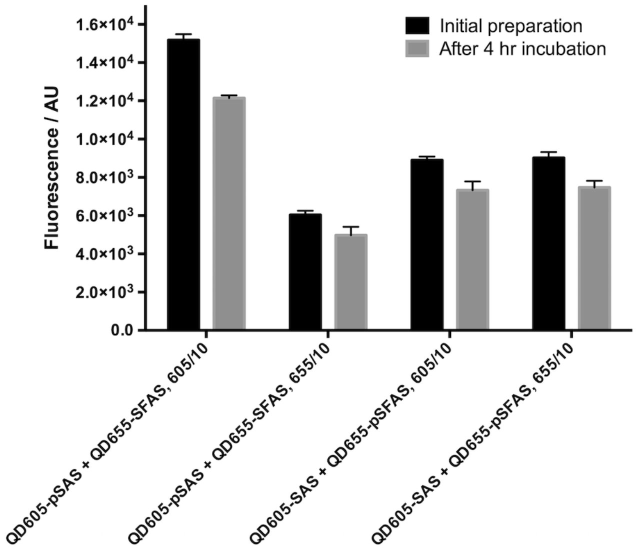

Figure S13. Ligand exchange test between QD605 and QD655 in 10 mM HEPES buffer (pH = 7.5). Detection was done by using a 605/10 and a 655/10 emission filter with 250 µs delay time and 1 ms integration time. Gain for 605/10 was 135 and that for 655/10 was 150 on the microplate reader. Conjugate concentration was 10 nM and that of Tb^{3+} was 10 µM. The labeled conjugate in each sample was incubated for 1h at room temperature before mixed with the other unlabeled QD.

As shown in Figure S13, one of the QD was labeled with phosphopeptide while the other one was labeled with orthogonal unphosphopeptide at room temperature for 1 hr. Mixed QD showed no visible ligand exchange indicated by the LRET emission after 4 hrs incubation. The slight decrease in signal intensity after 4 hrs incubation was universal among all samples, therefore it should be considered as instrumental offset between measurements rather than ligand exchange, the latter of which should cause signal decrease for the QDs labeled with phosphopeptides and signal increase for the QD labeled with unphosphopeptides.

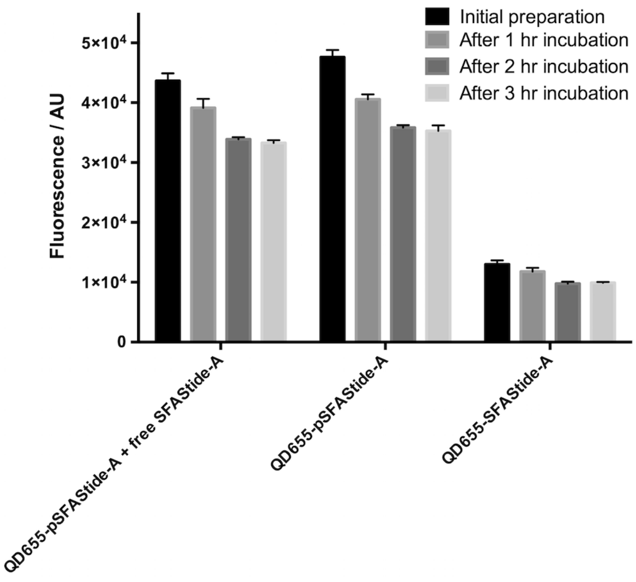

Figure S14. Ligand competition test using QD655-pSFAStide-A and free SFAStide in 10 mM HEPES buffer (pH = 7.5). Conjugates were prepared by 20 nM QD and 4 μM peptides (1:200 ratio) to ensure saturated QD labeling before the competing peptides were added. Detection was done by using a 655/10 emission filter with 250 μs delay time and 1 ms integration time. Gain for 655/10 was 150 on the microplate reader. Conjugate concentration was 20 nM and that of Tb^{3+} was 20 μM. The concentration of free SFAStide-A used in the first sample was 4 μM.

A ligand competition test was also performed by incubation 20 nM pre-formed QD655-pSFAStide-A conjugate with 4 μM free SFAStide-A (Figure S14). When comparing its signal with positive (QD655-pSFAStide-A only) and negative (QD655-SFAStide-A only) controls at selected time points, no visible ligand exchange (i.e. significant formation of QD655-SFAStide-A conjugate) was indicated by the LRET emission after 1, 2, or 3 hrs of incubation. The variance in signal intensity at different time points was universal among all samples, therefore it was likely to arise from instrumental offset between measurements rather than ligand exchange, the latter of which would cause decreased QD655 LRET emission for the exchange sample when compared with the positive control.

Delay time and signal to background ratio: Due to the strong residual QD steady state luminescence and background time-resolved emission (Figure S12), a short delay time for time-resolved detection may not achieve best signal to background ratio.

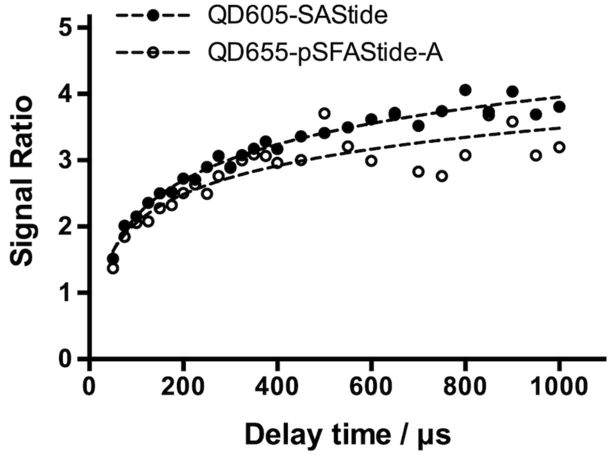

Figure S15. Signal to background ratio increased with the increase of delay time in time-resolved detection. This ratio was calculated as the signal intensity ratio of QD-phosphobiosensor to QD-unphosphobiosensor. All conjugates were prepared in 10 mM HEPES buffer (pH = 7.5) with 10 µM Tb^{3+}. Conjugate concentration was 10 nM (QD:peptide ratio = 1:100). Detection was done by using a 605/10 and a 655/10 emission filter with increasing delay time and 1 ms integration time. Gain for 605/10 was 135 and that for 655/10 was 150 on the microplate reader.

Longer delay time resulted in higher signal to background (QD-phosphobiosensor/QD-unphosphobiosensor signal ratio) ratio (Figure S15). However, longer delay time also results in lower signal intensity, therefore when developing assays based on the work reported here, delay time needs to be optimized to find a compromise between background and signal, given the specific QD/peptide/Tb^{3+} ratio and the concentrations of all components in the reaction.

Calibration curves: In order to mimic the experimental condition of multiplexed kinase assay (as discussed in Figure S11), a calibration curve for QD605-SAStide was established in kinase reaction buffer with the existence of 100% unphosphorylated QD655-SFAStide-A (Figure S16a) and vice versa (Figure S16b). Multiplexed kinase assays (Figure 4) were quantified using their corresponding calibration curves.

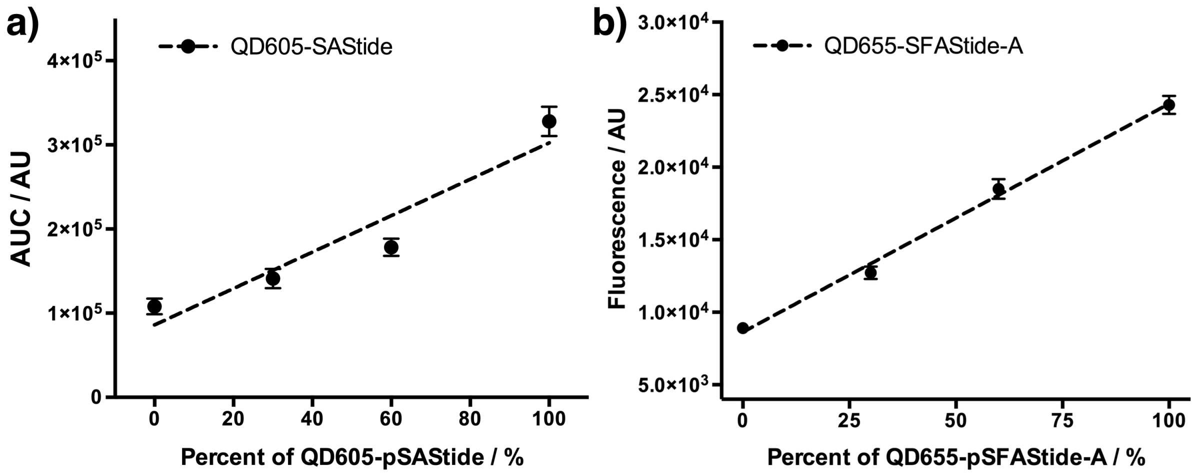

Figure S16. Calibration curves for multiplexed kinase assays were established by preparing samples with 15 nM kinase, 50 nM QDs, 100 µM ATP, 10 mM Mg^{2+}, 0.2 µg/µL BSA, and 5 µM peptides in which the portion of phosphorylated peptide was increasing, in 25 mM HEPES buffer. Aliquots of samples (40 µL) were diluted to in detection buffer (40 µL 6 M urea, 10 µL 1 M NaCl, 10 µL 1 mM Tb^{3+}) followed by spectral detection. Time-resolved spectra of a) were recorded on a Synergy 4 microplate reader with 400 µs delay and 1000 µs integration, while those of b) were recorded with 250 µs delay. Conjugate concentration was 20 nM (QD to peptide ratio = 1:100). Calibration curves were prepared by quantifying area under QD605 emission peak using monochromator for a), and quantifying readings from a 655/10 emission filter for b), demonstrating the feasibility of both methods.

Calculations: The Z' factor, signal window (SW), and coefficient of variation (CV) were calculated as described below using filter-based measurement. High-throughput screening (HTS) compatibility usually requires Z' > 0.5 and SW > 2[1].

Table S1. Parameters calculated from QD605-SAStide (10 nM) calibration standards

Parameters	0% [a)]	25%	50%	75%	100%
Z'	N/A	0.649	0.849	0.890	0.910
SW	N/A	12.584	39.316	41.683	42.635
CV	0.030	0.018	0.014	0.019	0.024

[a)]The percentage of phospho-conjugates.

Table S2. Parameters calculated from QD655-SFAStide-A (10 nM) calibration standards

Parameters	0% [a)]	25%	50%	75%	100%
Z'	N/A	0.529	0.789	0.806	0.943
SW	N/A	5.625	20.260	17.051	152.281
CV	0.038	0.042	0.027	0.045	0.007

[a)]The percentage of phospho-conjugates.

In many real-world cases, kinase assays are performed with endpoints that achieve less than 20% phosphorylated product (in order to achieve optimal Michaelis-Menten kinetics). In order to achieve sufficient signal intensity at lower % phosphorylation for the assay reported here, one can simply adjust the concentration and ratio(s) of assay reagents to achieve optimal HTS parameters. As shown in Table S3, QD655-SFAStide-A conjugates were prepared in 1:200 QD:peptide ratio, and 20 nM or 40 nM of mixed conjugates in the kinase reaction buffer described above were used to test HTS parameters. HTS compatible performance metrics were still achieved with 10% phosphorylation at nanomolar concentration (e.g. 40 nM, Table S3).

Table S3. Parameters calculated from low percent of QD655-pSFAStide-A calibration standards

Parameters	0% (20 nM)[a)]	10% (20 nM)	20% (20 nM)	0% (40 nM)	10% (40 nM)	20% (40 nM)
Z'	N/A	< 0.5	0.667	N/A	0.595	0.712
SW	N/A	< 2	20.996	N/A	9.662	14.109
CV	0.038	0.028	0.010	0.027	0.019	0.023

[a)]The percentage of phospho-conjugate. Concentration of conjugate is indicated in the brackets.

The Z' factor was calculated according to the following equation:

$$Z' = \frac{(\mu_p - \frac{3\sigma_p}{\sqrt{n}}) - (\mu_n + \frac{3\sigma_n}{\sqrt{n}})}{\mu_p - \mu_n}$$

The signal window (SW) was calculated according to the following equation:

$$SW = \frac{(\mu_p - \frac{3\sigma_p}{\sqrt{n}}) - (\mu_n + \frac{3\sigma_n}{\sqrt{n}})}{\frac{\sigma_p}{\sqrt{n}}}$$

In both equations, n is the number of replicates of measurements. μ_p and μ_n are the average signal of the positive (phospho-conjugates) and negative (unphospho-conjugates) controls, respectively. σ_p and σ_n are the standard deviation of the corresponding values.

[1] J. H. Zhang, *J. Biomol. Screen.* **1999**, *4*, 67.
